# A cross-sectional study of psychopathy and khat abuse among prisoners in the correctional institution in Jimma, Ethiopia

**DOI:** 10.1371/journal.pone.0227405

**Published:** 2020-01-16

**Authors:** Yimenu Yitayih, Matiwos Soboka, Elias Tesfaye, Mubarek Abera, Almaz Mamaru, Kristina Adorjan

**Affiliations:** 1 Department of Psychiatry, Faculty of Medical Sciences, Jimma University, Jimma, Ethiopia; 2 Department of Psychiatry and Psychotherapy, Medical Center of the University of Munich, Munich, Germany; 3 Institute of Psychiatric Phenomics and Genomics (IPPG), Medical Center of the University of Munich, Munich, Germany; 4 Center for International Health, Ludwig-Maximilians-Universität, Munich, Germany; University of Toronto, CANADA

## Abstract

**Background:**

Khat abuse and psychopathy are both strongly related to criminal activity. Higher rates of substance use in people with psychopathy are hypothesized to be related to psychopathic personality traits, which include high sensation seeking, low conscientiousness and neuroticism, impulsivity, and irresponsibility. Little is known, however, about the association between psychopathy and khat abuse among prisoners in Ethiopia. Therefore, we evaluated the presence of these two factors in prisoners in the correctional institution in Jimma, Southwest Ethiopia.

**Materials and methods:**

We used a cross-sectional study design to collect data from 336 prisoners from June 5 to July 5, 2017. Study participants were selected by a systematic random sampling technique. Khat abuse was assessed with the Drug Abuse Screening Tool and psychopathy with the Psychopathy Checklist: Screening Version. We also assessed nicotine dependence with the Fagerstrom Test for Nicotine Dependence; alcohol use disorder, with the alcohol use disorder identification test; adverse traumatic life events, with the Life Events Checklist; and social support, with the Oslo 3-Item Social Support Scale. Data were entered into EpiData version 3.1 and analyzed in bivariate and multivariable logistic regression models. Variables with a P value < 0.05 in the final fitted model were declared to be significantly associated with the outcome variable.

**Results:**

The overall prevalence of lifetime khat use was 59.9%, and the prevalence of khat abuse in prisoners with psychopathy was 78.0%. Prisoners with psychopathy had a three times higher odds ratio of abusing khat than those without psychopathy (AOR = 3.00 [1.17–7.67]). Among the confounders, a family history of substance use (AOR = 2.50 [1.45–4.31]), poor support (AOR = 2.28 [1.11–4.67]), alcohol use disorder (AOR = 7.78 [4.16–14.53]), and suicidal ideation and suicide attempts (AOR = 2.26 [1.45–4.31]) were also positively associated with khat abuse.

**Conclusions:**

The prevalence of khat abuse was higher in prisoners with possible or probable psychopathy.

## Introduction

Khat (Catha edulis) is a plant whose leaves and branches are chewed for their psychomotor stimulant effects [1]. Khat has numerous names, including kat, qat, qaad, and miraa, and contains different alkaloids, including cathinone, cathine, and norephedrine. Cathinone is the main active component and causes excitement, loss of appetite, and psychotic symptoms [2]. The prevalence of khat chewing is related to sociocultural habits, the accessibility of khat, and the enforcement of laws. An estimated 10 million people worldwide chew khat leaves daily [3]. According to the 2016 Ethiopia Demographic and Health Survey, 12% of women and 27% of men have reported having ever chewed chat [4].

Psychopathy is a personality disorder with interpersonal, emotional, and antisocial features [5, 6] that is characterized by abnormal affective, interpersonal, and behavioral functioning. Psychopathic traits include emotional deficits, such as a profound inability to experience empathy and remorse; additional features include behavioral problems, such as impulsivity, stimulation seeking, and instrumental aggression [5, 6]. These traits tend to co-occur with a number of problem behaviors, including substance misuse [7], and some studies show that they may lead to increased substance use [8, 9]. Research has also indicated that the motivating factor for substance use is linked to psychopathic personality traits, including high sensation seeking, low conscientiousness and neuroticism, impulsivity, and irresponsibility [8, 9].

Khat abuse and psychopathy are both strongly related to criminal activity. Individuals with psychopathy are among the most dangerous and chronic offenders, as evidenced by high re-offending rates [10, 11]. Psychopathic personality is an important construct that has been linked to criminal behavior and problematic substance use [12], and substance use greatly increases the likelihood that psychopathic individuals will engage in serious or violent criminal activity [12]. Indeed, a large-scale study of aggression and offending found that the best predictor of violence was psychopathic traits in conjunction with substance use [12]. Relative to non-psychopathic offenders, people with psychopathic traits are more likely to have a diagnosis of substance abuse or dependence and of poly-substance use [12]. Despite the relevance of psychopathy and khat use for criminal behavior, little is known about their prevalence among prisoners in Ethiopia.

The data presented in this paper were obtained as part of a study on substance use disorder and associated factors among prisoners in the correctional institution in Jimma, Southwest Ethiopia [13]. The findings on an association between psychopathy and substance use disorders in general have been published elsewhere [13]. Here, we present detailed analyses of the association between psychopathy and khat abuse in this population. In line with the results of previous studies [14, 15], we hypothesized that psychopathy is significantly associated with khat abuse.

## Materials and methods

### Study design and setting

We conducted a cross-sectional study in the correctional institution in Jimma, Southwest Ethiopia, from June 5 to July 5, 2017. The facility serves the Oromia, Southern Nations and Nationalities People’s, and Gambella regions and houses about 1460 prisoners (at the time of the study: 1418 men and 42 women). The prison population includes offenders on remand and people convicted to a limited or lifelong sentence.

### Sample size

To calculate the sample size (n), we used the single population proportion formula n = (Z_α/2_)^2^ * P (1-P) / d^2^ with a prevalence (P) of 50%, i.e. 0.5 (because no similar study has been performed in a prison population in Ethiopia), a 95% confidence interval (CI) (Zα/2 = 1.96), and a type I error of 5% (D, 0.05). The formula resulted in a sample size of 384. Because the population size was <10,000, we used the finite population correction formula with the calculated sample size of 384 and the total population of 1460 to give n_f_ = 305. Thus, assuming a 10% non-response rate the final sample size was 336.

### Sampling technique

We selected study participants by a systematic random sampling technique. The total number of prisoners was obtained from the prison administrator and used as the sampling frame. Prisoners who were unable to communicate were excluded from study, so that 1460 prisoners were eligible for the study. The sampling interval was four (1460/336). First, we randomly selected one participant from the first four prisoners listed in the registration book, where prisoners are recorded in the order of their arrival at the prison.

Then, we used the systematic random sampling technique to select one prisoner from every subsequent group of four until the required sample size was reached (n = 336).

### Data collection procedures

We performed a pre-test on 5% of the prisoners in the Agaro prison, 45 kilometers from Jimma, and subsequently corrected some ambiguous words on the data collection questionnaire on the basis of the responses in the pre-test. In the main study, data were collected by five postgraduate students in mental health. All data collectors were given training on the study objectives, data collection methods, and assessment tools and on maintaining confidentiality, obtaining informed consent and handling ethical issues. The five students were supervised by two Masters of Science in Public Health students and the principal investigator. Questionnaires were checked for completeness, and data were entered into a computer for further processing.

We assessed khat abuse with the Drug Abuse Screening Test (DAST), a 10-item, self-administered questionnaire. The DAST was developed to screen for the use of drugs, including amphetamine, which has a similar chemical structure and similar biochemical effects to khat; a score ≥3 on the DAST indicates khat abuse [16]. The reliability of the DAST in this study was 0.88 (Cronbach’s α). We also assessed lifetime khat use by asking whether respondents had ever used khat.

Psychopathy was measured with the Psychopathy Checklist: Screening Version (PCL: SV). The PCL: SV contains 12 items that assess typical features of a psychopathy life history, i.e. features of criminal, social, occupational, and childhood family environments; items are scored as 0 = not present, 1 = borderline present, and 2 = present. The scores can be interpreted categorically: A score ≥18 is taken to indicate probable psychopathy; and a score <18 but ≥13, possible psychopathy [17]. The sensitivity of the PCL-SV is 0.94 and the specificity 0.85 [17], and the reliability in this study was Cronbach’s α = 0.86.

We assessed the participants’ PCL: SV score by both interview and a file review. The PCL: SV was administered by postgraduate students in mental health, who had been trained by a psychiatrist trained in the use of the instrument.

Data collectors conducted the PCL: SV assessment interviews after performing test assessment interviews in the presence of a trainer to establish norms for scoring individual PCL: SV items. In addition, the trainer also supervised the data collectors during the study.

In addition to examining psychopathy and khat abuse, we assessed the presence of alcohol use disorders (AUDs) with the World Health Organization’s Alcohol Use Disorders Identification Test (AUDIT) [15]. An AUDIT score ≥8 was taken to indicate an AUD. The sensitivity and specificity of AUDIT for AUD are 0.90 and 0.80, respectively [18], and the reliability of AUDIT in this study was 0.87 (Cronbach’s α). Also, we assessed adverse traumatic life events with the Life Events Checklist (LEC); a traumatic life experience was defined as experiencing at least one traumatic event. The LEC was developed by the National Center for Posttraumatic Stress Disorder to aid in the detection of posttraumatic stress disorder (PTSD); it has been widely used in cross-cultural settings and is predictive of AUD, anxiety, depression, and PTSD [19]. Nicotine dependence was assessed by the Fagerstrom Test for Nicotine Dependence (FTND; score ≥1 indicates nicotine dependence) [20]. The reliability of the FTND in this study was 0.80 (Cronbach’s α). We also assessed social support with the Oslo 3-item Social Support Scale [21]. Finally, we used a questionnaire to assess the following potential explanatory variables for substance use disorder: socioeconomic factors (age, sex, marital status, ethnicity, religion, educational status, occupation, income); environmental factors (family history of substance use, social support, immigration history); behavioral and mental health factors (previous known mental illness, perception that substance use does not impair health, start of substance use at an early age, chronic physical illness, suicidal ideation and attempts); and criminal factors (previous arrests, previous substance-related offences, type of crime, committed a crime under the influence of a substance).

### Data analysis

After the tests and questionnaires had been checked for completeness, data were entered into EpiData Version 3.1 and then exported to the Statistical Package for Social Science version 21.0 for further analysis. Descriptive statistics, such as frequencies and medians, were computed, and bivariate and multivariable analyses were used to identify factors associated with the outcome variables.

All variables associated with khat abuse in the bivariate logistic regression with a P value < 0.25 were entered together into the multivariable logistic regression by default (enter method) to control for potential confounders. Variables with P value < 0.05 were declared to be associated with khat abuse in the final model. An odds ratio with a 95% confidence interval (95% CI) was calculated to assess the level of association and statistical significance.

### Ethical considerations

The study protocol was approved by the Research Ethics and Approval Committee of the Jimma University Institute of Health, and the study was performed in accordance with the Declaration of Helsinki. Verbal informed consent was obtained from participants. Prisoners were assured of confidentiality, and they were informed that participation was voluntary and that they could withdraw at any time during the interview without giving a reason for doing so. They were told that their agreement or refusal to participate in the study would not affect their prison sentence or the possibility of parole. Participants were told that selection for participation in the study was random and that they had the right not to respond to questions that they were not comfortable with and the right to ask questions. The interview was conducted in a private room, and no other individuals were present during the interview. After data entry was complete, the questionnaires were kept securely locked away. Study participants found to have psychopathy and khat abuse were referred to Jimma University Medical Centre.

## Results

### Socio-demographic characteristics

A total of 329 prisoners participated in the study. The response rate was 97.9%; of the 336 prisoners invited to participate, n = 7 (2.1%) declined because they were unwilling to be interviewed about their khat use histories. The median age of participants was 26 years (interquartile range [IQR] 14). Most of the participants had been residing in urban areas before imprisonment and were unmarried. The most common religion was Muslim. Detailed information on socio-demographic characteristics is given in Table 1.

**Table 1:** Socio-demographic characteristics of prisoners in the correctional institution in Jimma, Southwest Ethiopia, June-July 2017 (n = 329).

Variable	Category	n	Percentage (%)
Sex	Male	307	93.3
	Female	22	6.7
Age	<30	219	66.6
	≥ 30	110	33.4
Residential setting	Rural	106	32.2
	Urban	223	67.8
Educational status	No formal education	27	8.2
	Primary education	178	54.1
	Secondary education	94	28.6
	Tertiary education	30	9.1
Religion	Muslim	181	55.0
	Orthodox	97	29.5
	Protestant	38	11.6
	Catholic	13	4.0
Marital status	Married	124	37.7
	Unmarried	205	62.3
Occupation	Employed	134	40.7
	Unemployed	36	10.9
	Farmer	99	30.1
	Student	43	13.1
	Other**	17	5.2
Average monthly income (birr)	<1200	192	58.4
	≥1200	137	41.6

Other

**: retired or homemaker

### Prevalence of psychopathy and khat abuse

Possible psychopathy was present in 26/329 participants (7.9%; PCL: SV score ≥13 but <18); and probable psychopathy, in 15/329 (4.6%; PCL: SV score ≥18). Psychopathy symptoms are shown in Table 2. In the total sample, the prevalence of lifetime khat use was 197/329 (59.9%); and of khat abuse, 138/329 (41.9%). Among the participants with possible or probable psychopathy, 32/41 (78.0%) had a history of khat abuse, and among prisoners with khat abuse, 21/138 (15.2%) had possible psychopathy and 11/138 (8.0%) had probable psychopathy. The most common psychopathic traits were pathological liar (18/138; 13.0%) and a history of juvenile delinquency (14/138; 10.1%).

**Table 2:** Correlations of khat abuse, psychopathic traits among prisoners, Ethiopia, 2017.

Psychopathic traits		Khat abuse
Glib and superficial charm	Pearson Correlation	.202**
	Sig. (2-tailed)	.000
	N	329
Grandiose self-worth	Pearson Correlation	.113*
	Sig. (2-tailed)	.041
	N	329
Pathological lying	Pearson Correlation	.259**
	Sig. (2-tailed)	.000
	N	329
Lack of remorse or guilt	Pearson Correlation	.208**
	Sig. (2-tailed)	.000
	N	329
Callousness and lack of empathy	Pearson Correlation	.234**
	Sig. (2-tailed)	.000
	N	329
Early behavior problems	Pearson Correlation	.254**
	Sig. (2-tailed)	.000
	N	329
lack of realistic long-term goals	Pearson Correlation	.264**
	Sig. (2-tailed)	.000
	N	329
Impulsivity	Pearson Correlation	.270**
	Sig. (2-tailed)	.000
	N	329
Irresponsibility	Pearson Correlation	.273**
	Sig. (2-tailed)	.000
	N	329
Failure to accept responsibility for own actions	Pearson Correlation	.273**
	Sig. (2-tailed)	.000
	N	329
Juvenile delinquency	Pearson Correlation	.216**
	Sig. (2-tailed)	.000
	N	329
Adult anti-social	Pearson Correlation	.249**
	Sig. (2-tailed)	.000
	N	329

### Types of crimes committed

The most common crimes among prisoners with possible or probable psychopathy were murder 13/41 (31.7%) and assault 13/41 (31.7%) (see Table 3).

**Table 3:** Types of crime committed by prisoners with psychopathy (n = 329) in the correctional institution in Jimma, Southwest Ethiopia, in June-July 2017.

Type of offence	Psychopathy	
	No (%)	Yes (%)
Robbery	12 (4.2)	3 (7.3)
Rape	29 (10.1)	3 (7.3)
Murder	70 (24.3)	13(31.7)
Theft	73 (25.3)	8 (19.5)
Assault	90 (31.3)	13 (31.7)
Others*	14 (4.9)	1 (2.5)
Total	288 (100)	41 (100)

* Political offence or offence related to forest destruction

### Factors associated with khat abuse

The bivariate analysis found that various socio-demographic, behavioral, mental health, environmental, and criminal factors were associated with khat abuse, as follows: male sex (crude odds ratio [COR]: 2.01; 95% CI: 0.77, 5.128; p = 0.156), living in an urban setting (COR: 1.64; 95% CI: 1.01, 2.65), p = 0.044), psychopathy, multiple traumatic life events, suicidal ideation and suicide attempts, family history of substance use, poor social support, immigration history, and previous history of imprisonment (see Table 4). These variables were included in the multivariable analysis (see Table 5).

**Table 4:** Bivariate analysis of behavioral, mental health, environmental, and criminal factors among prisoners in the correctional institution in Jimma, Southwest Ethiopia, June-July 2017 (n = 329).

Variable		Khat abuse		COR (95% CI)	P value
		No (%)	Yes (%)		
Sex	Male	175 (57.0)	132 (43.0)	2.01 (0.77–5.28)	0.156*
	Female	16 (72.7)	6 (27.3)	Reference value	
Residential setting	Rural	70 (66.0)	36 (34.0)	Reference value	
	Urban	121 (54.3)	102 (45.7)	1.64 (1.01–2.65)	0.044*
Psychopathy	No	182 (63.2)	106 (36.8)	Reference value	
	Yes	9 (22.0)	32 (78.0)	6.11 (2.81–13.28)	0.001*
Adverse traumatic life event	No exposure to traumatic life event	77 (64.2)	43 (35.8)	Reference value	
	One traumatic life event	38 (60.3)	25 (39.7)	1.18 (0.63–2.21)	0.609
	Multiple traumatic life events	76 (52.1)	70 (47.9)	1.65 (1.01–2.71)	0.047*
Mental illness	No	176 (59.1)	122 (40.9)	Reference value	
	Yes	15 (48.4)	16 (51.6)	1.54 (0.73–3.23)	0.254
Social support	Poor	88 (47.8)	96 (52.2)	2.494 (1.38–4.49)	0.002*
	Moderate	55 (72.4)	21 (27.6)	0.87 (0.43–1.79)	0.710
	Strong	48 (69.6)	21 (30.4)	Reference value	
Immigration	No	162 (60.0)	108 (40.0)	Reference value	
	Yes	29 (49.2)	30 (50.8)	1.55 (0.88–2.73)	0.128*
Previous imprisonment	No	180 (59.8)	121 (40.2)	Reference value	
	Yes	11 (39.3)	17 (60.7)	2.30 (1.04–5.08)	0.040*
Family history of substance use	No	132 (68.4)	61 (31.6)	Reference value	
	Yes	59 (43.4)	77 (56.6)	2.824 (1.79–4.45)	0.001*
Suicidal ideation and suicide attempts	No	152 (62.6)	91 (37.4)	Reference value	
	Yes	39 (45.3)	47 (54.7)	2.01 (1.22–3.31)	0.006*
Chronic physical illness	No	161 (58.3)	115 (41.7)	Reference value	
	Yes	30 (56.6)	23 (43.4)	1.07 (0.59–1.94)	0.815
Perceived that substance use did not affect health	No	83 (58.5)	59 (41.5)	Reference value	
	Yes	108 (57.8)	79 (42.2)	1.03 (0.66–1.60)	0.899

Reference value: In the analysis, this variable indicated lower likelihood of alcohol use; coded as zero in SPSS logistic regression

*Identified as factors for multivariable logistic regression analysis (p<0.25)

COR: Crude odds ratio

95% CI: 95% confidence interval

**Table 5:** Multivariable logistic regression analysis of independent predictors of khat abuse among prisoners (n = 329) in the correctional institution in Jimma, Southwest Ethiopia, in June-July 2017.

Variable		Khat abuse		AOR (95% CI)
		No (%)	Yes (%)	
Psychopathy	No	182 (63.2)	106 (36.8)	Reference value
	Yes	9 (22.0)	32 (78.0)	3.00 (1.17–7.67)
Social support	Poor support	112 (60.9)	72 (39.1)	2.28 (1.11–4.67)
	Moderate support	49 (64.5)	27 (35.5)	0.61 (0.25–1.52)
	Strong support	49 (71.0)	20 (29.0)	Reference value
Family history of substance use	No	135 (69.9)	58 (30.1)	Reference value
	Yes	75 (55.1)	61 (44.9)	2.50 (1.45–4.31)
Suicidal ideation and suicide attempts	No	152 (62.6)	91 (37.4)	Reference value
	Yes	39 (45.3)	47 (54.7)	2.26 (1.23–4.17)
Alcohol use disorder	No	159 (75.7)	51 (24.3)	Reference value
	Yes	32 (26.9)	87 (73.1)	7.78 (4.16–14.53)

AOR: adjusted odds ratio; 95% CI: 95% confidence interval

Reference value: In the analysis, this variable indicated a lower likelihood of khat abuse; coded as zero in SPSS logistic regression

The multivariable analysis indicated that the following variables were associated with higher odds of having khat abuse: psychopathy, family history of substance use, poor social support, AUD, and suicidal ideation and suicide attempts (Table 5).

## Discussion

In this study, we assessed the co-morbidity of psychopathy and khat abuse among prisoners in the correctional institution in Jimma, Southwest Ethiopia. We found that the prevalence of khat abuse in the 12 months before imprisonment was higher in prisoners with possible or probable psychopathy than in those with no psychopathy.

Psychopathy was one of the factors significantly associated with khat abuse in this study, and participants with possible or probable psychopathy had three times higher odds of khat abuse than those with no psychopathy.

This finding is in line with a study performed in England and Wales, which found that psychopathy is a factor for substance use [22]. The association between psychopathy and substance use is hypothesized to be related to psychopathic personality traits, such as irresponsibility, impulsivity, risk taking, and antisocial behavior [22]. Mental health services in Ethiopia are primarily centralized around Amanuel Mental Specialized Hospital, in the capital city. Psychiatric care in communities is severely affected due to unequal distribution of resources, problems of access to services in remote locations and stigma against people with mental illness. There is a crucial need to improve the delivery of mental health services, particularly those related to the control and prevention of mental illness and substance abuse.

Our findings have implications both for future research on the etiology of offending among people who abuse khat and have AUD and for recognizing ways to prevent offending in this group. The presence from an early age of behavioral problems can escalate into delinquency, criminality, and substance use. We hypothesize that among life-course–persistent offenders, the antecedents of psychopathic traits emerge very early in life. Future research should be designed to more adequately measure the first signs of psychopathic traits, neurobiological correlates, and the psychosocial factors that act to strengthen and weaken the traits at critical times during development. Furthermore, the roles played by intoxication and substance misuse, abuse, and dependence in offending need to be understood.

The findings of the present study also have implications for intervention. People dually diagnosed with psychopathy and substance use present unique challenges. Although they may only represent a small portion of individuals in substance use treatment programs, the presence of substance abuse and personality pathology requires a disproportionate amount of staff attention, time, and money [23]. Furthermore, these people’s motivation for therapeutic change is often weak because people with psychopathy and a substance use disorder rarely present for treatment voluntarily. Prison settings thus represent a golden opportunity to administer clinical interventions in people with psychopathy, khat abuse, and AUD [24].

Once people with psychopathy are in treatment, core psychopathic traits, such as a lack of empathy, an inability to form close personal relationships, and manipulative and callous behaviors, are barriers to successful therapeutic interventions. Some researchers believe that despite good compliance with therapy and reported therapeutic improvement in correctional settings people with psychopathy simply use what they learn in therapy to supplement their criminal versatility and skill, which explains the well-known negative outcomes of therapy in this population [25].

By treating this population on the basis of their characteristics, we can change both the individuals’ outcomes and the investment that the research community has made in developing new and more effective treatment options for people with psychopathy who misuse substances.

Our study had some limitations. Social desirability bias may have led prisoners to underestimate or underreport khat use. Recall bias may also have been a problem because the data were collected by self-report during an interview and prisoners were reporting on their khat use before being imprisoned, so they may have underestimated khat abuse. The DAST and PCL: SV were not validated in our population, although these instruments have been shown to be useful in screening for amphetamine and psychopathy, respectively, across cultures. We did not check the validity of participants’ responses, for example by performing laboratory tests to detect substances. In addition, we collected data on prisoners only and did not compare the prevalence of khat abuse with a non-prison population. Last, the study used a cross-sectional design and therefore was unable to show a cause and effect relationship between psychopathy and khat abuse.

## Conclusions

Our study found that the prevalence of khat abuse in the Jimma correctional institution was higher in prisoners with possible or probable psychopathy, i.e. prisoners with possible or probable psychopathy had a higher odds ratio for khat abuse than those with no psychopathy. A family history of substance use, poor social support, AUD, and suicidal ideation and suicide attempts were also associated with khat abuse.

The study highlights the need to strengthen addiction health services in the correctional institution in Jimma and to design and implement long-term management plans for recovery and rehabilitation from khat abuse. There was a high prevalence of traumatic life event exposure among the prisoners in this study, so we recommend that researchers evalaute prisoners for posttraumatic stress disorder. Further research is needed to determine whether the findings of this study apply to other correctional institutions in Ethiopia and perhaps elsewhere.
